# Immunodetection of Porcine Red Blood Cell Containing Food Ingredients Using a Porcine-Hemoglobin-Specific Monoclonal Antibody

**DOI:** 10.3390/foods6110101

**Published:** 2017-11-20

**Authors:** Jack A. Ofori, Yun-Hwa P. Hsieh

**Affiliations:** Department of Nutrition, Food and Exercise Sciences, 420 Sandels Building, Florida State University, Tallahassee, FL 32306-1493, USA; papajackx@yahoo.co.uk

**Keywords:** monoclonal antibody, porcine blood, ingredients, red blood cells, hemoglobin, ELISA

## Abstract

Monoclonal antibody (mAb) 24C12-E7 has been found to bind to a 12 kDa antigenic protein in the red blood cell (RBC) of porcine blood. The purpose of this study was to determine the identity of this 12 kDa protein and consequently examine its potential as a marker for monitoring porcine RBC-containing ingredients (PRBCIs) in foods. Proteomic techniques identified the 12 kDa antigenic protein to be a monomer of the tetrameric hemoglobin molecule. Further heat-processing of spray-dried PRBCIs diminishes its detectability. Whereas mAb 24C12-E7-based indirect enzyme-linked immunosorbent assay (iELISA) could detect 1% (*v*/*v*) or less of PRBCIs in raw and cooked ground meats (beef, pork and chicken), the detection limits were 3 to 30 times higher for spiked cooked beef and pork. The assay is effective for monitoring the presence of PRBCIs in foods to protect the billions of people that avoid consuming blood. In situations where these PRBCIs are present as ingredients in foods that have undergone further heat processing, the assay, however, may not be as sensitive depending on the types of sample matrix, types of PRBCIs and the level of inclusion.

## 1. Introduction

The use of blood-derived proteins mainly produced from porcine or bovine origin as food ingredients is increasing in both conventional and unconventional food products and is a source of worry for those that avoid consuming blood for various reasons. Consumer concerns are heightened by the fact that these protein ingredients are often declared by their brand names on the food labels and hence unknown to the consumer that they are blood-derived. To protect the interest of the particular sector of consumers, efforts have been made towards developing analytical methods for monitoring the presence of these blood-derived ingredients in dietary products. However, the task is arduous as these proteins are diverse in nature in the sense that they could be produced from whole blood, the plasma or red blood cell (RBC) fraction of the blood, or hydrolyzed isolated blood proteins.

From a panel of previously developed monoclonal antibodies (mAbs) that bind to bovine blood, either raw or heat-treated (100 °C for 15 min or 121 °C at 1.2 bar for 15 min) [1], Subsequently, a sandwich enzyme-linked immunorsobent assay (sELISA) was developed for detecting bovine plasma-derived food ingredients [2] and a competitive ELISA (cELISA) for detecting bovine RBC-derived food ingredients [3]. However, complementary methods to detect porcine-derived blood ingredients are still lacking. The only work performed apart from our laboratory was the work by Raja Nhari and others [4] who reported of mAbs that they have developed against heat-treated porcine blood. They mentioned that two mAbs found to be bind to a 60 kDa protein in porcine plasma could be useful for the detection of porcine plasma in processed food. However, judging from the very low absorbance readings (<0.06) obtained for the mAbs against blood samples, the usefulness of this assay in detecting blood in processed food is dubious. In addition, given the diverse nature of these blood proteins, actual samples would have to be tested to guarantee the usefulness of the assay. In the case of food ingredients derived from porcine red blood cells, there is no study reported in the literature on the subject.

Recently, we have raised porcine-blood-specific mAbs with the intent to use them as probes in immunoassays either individually or in various combinations for monitoring porcine blood-derived food ingredients. Two of these porcine blood antibodies that bind cooperatively to a 90 kDa antigenic protein in the plasma fraction of porcine blood, when used in sELISA, have been found effective in detecting porcine plasma-derived ingredients in foods and dietary supplements [5] Among the same panel of developed mAbs that bind to porcine blood, mAb 24C12-E7 was found to be porcine blood-specific and to react with a 12 kDa antigenic protein in the RBC fraction of porcine blood. In a previous study where we raised bovine-blood-specific antibodies for the purpose of probing the presence of bovine blood material in both feed and food using immunoassay [1], further studies revealed that the 12 kDa antigenic protein recognized by one of the antibodies, mAb Bb1H9, was a monomer of the tetrameric bovine hemoglobin molecule [6]. Based on this hind sight information, we anticipated this 12 kDa protein recognized by the porcine-blood-specific mAb 24C12-E7 likely to be a peptide of porcine hemoglobin. Thus, the objectives of this study were to (1) establish the identity of this 12 kDa antigenic protein recognized by mAb 24C12-E7; and (2) consequently determine its potential as a suitable marker for the detection of porcine RBC-derived food ingredients using indirect non-competitive ELISA (iELISA) probed by mAb 24C12-E7.

## 2. Materials and Methods 

Hydrogen peroxide, β-mercaptoethanol, isotyping kit (ISO-2 1 Kit), ABTS (2,2′-azino-bis 3 ethylbenzthiazoline-6-sulfonic acid), EZblue^TM^ gel staining reagent, and porcine hemoglobin were purchased from Sigma-Aldrich Co. (St. Louis, MO, USA). Alkaline phosphates (AP) conjugate substrate kit, Protein Assay Kit, goat anti-mouse IgG (H + L) AP (anti-Ig-AP) conjugate, Tris-buffered saline (TBS), 0.5 M Tris-HCl buffer (pH 6.8), 1.5 M Tris-HCl (pH 8.8), TEMED (*N*,*N*,*N*′,*N*′-tetra-methyl-ethylenediamine), Precision Plus Protein Kaleidoscope Standards, 30% acrylamide/bis solution, Tris/glycine buffer, Tris/glycine/SDS buffer, supported nitrocellulose membrane (0.2 μm), 7 cm 7 to 10 immobilized pH gradient (IPG) strip, rehydration/sample buffer, equilibration buffer I and II, and thick blot paper were purchased from Bio-Rad Laboratories Inc., Hercules, CA, USA. Egg albumin, bovine serum albumin (BSA) and all other chemicals were purchased from Fisher Scientific (Fair Lawn, NJ, USA). All chemicals and reagents were analytical grade and solutions were prepared using distilled deionized pure water (DD water) from a NANOpure DIamond ultrapure water system (Barnstead International, Dubuque, IA, USA).

Liquid samples of whole blood from pigs, cattle, donkey, horse, goat, sheep, rabbit, turkey, and chicken, porcine plasma, porcine serum and porcine red blood cells were purchased from Lampire Biological Laboratories, Pipersville, PA. Porcine gelatin and soy protein powders were obtained from GELITA USA Inc. (Sioux City, IA, USA) and SoyLink (Oskaloosa, IA, USA), respectively. Commercially produced porcine (porcine plasma powder (PPP), porcine Fibrimex^®^ powder (PFP), porcine hemoglobin powder (PHP), porcine hydrolyzed globin (PHG), Aprosan (APS), Aprothem (APT) Apropork (APP), and Aprored (APR)) and bovine (bovine plasma powder (BPP), bovine Fibrimex^®^ powder (BFP), bovine hemoglobin powder (BHP), bovine fibrinogen powder (BFGP) and Immunolin^®^ (ILN)) blood-derived food ingredients were obtained from Sonac BV, Eindhoven, Netherlands (PPP, PFP, PHP, PHG, BPP, BFP, BHP, and ILN) or from Proliant Inc., Barcelona, Spain (APP, APR, APS and APT) or from Proliant Inc., Ankeny, IA, USA. Nonfat dry milk was purchased from a local grocery store. Meat samples, including beef eye of round roast, pork loin, lamb shoulder, whole chicken, whole duck, whole goose, turkey breast, bison, and frozen dressed rabbit were purchased from a local supermarket. Horse meat was obtained from the College of Veterinary Medicine, Auburn University (Auburn, AL, USA). Deer, elk and African buffalo steak meats were provided by the Fats and Proteins Research Foundation (Bloomington, IL, USA).

### 2.1. Sample Preparation

#### 2.1.1. Extraction of Soluble Proteins from Animal Blood and Non-Blood Materials 

Soluble proteins were extracted from cooked (100 °C, 15 min) blood samples (whole blood, plasma, serum and RBCs), common food protein ingredients (soy powder, bovine gelatin, porcine gelatin, egg albumin and BSA) and meat samples (pork, beef, horse, elk, donkey, lamb, Bison, African buffalo, rabbit, turkey, chicken, goose, and duck) as previously described [1]. Extraction of soluble proteins from commercially produced porcine and bovine blood ingredients was performed as previously reported for commercially feedstuffs [1]. Raw blood samples were used as is. The protein extracts were stored at −20 °C until use.

#### 2.1.2. Spiked Sample Extracts 

To study the effect of matrix on the detectability of this 12 kDa antigenic protein, spiked samples were prepared as follows and probed with 24C12-E7 using iELISA. 

To 9.5 g of ground chicken in a sampling bag was added 0.5 g of porcine RBC-containing product (PHP, APS, APT or APR), and the mixture stirred thoroughly with a glass rod to obtain 5% *w*/*w* spiked ground chicken. Then, 50 mL of 10 mM phosphate buffered saline (PBS, pH 7.2) was added to the mixture in the sampling bag and the mixture was homogenized first by hand and then for another 1 min using a stomacher (Model Number STO 400, Tekmar Company, Cincinnati, OH, USA). The homogenized samples were then stored overnight at 4 °C after which it was centrifuged at 3220 *g* for 1 h at 4 °C (Eppendorf 5810R centrifuge, Brinkman Instruments Inc., Westbury, NY, USA) and then passed through Whatman # 1 filter paper to obtain 5% *w*/*w* spiked raw ground chicken extracts. Non-spiked raw chicken (0%) containing no added target analyte was similarly prepared. Lower levels of spiking (0.01%, 0.03%, 0.05%, 0.1%, 0.3%, 0.5%, 1% and 3% *v*/*v*) were obtained by diluting 5% *v*/*w* spiked samples with the appropriate amount of cooked chicken meat (0%) to ensure homogeneity. 

Another set of 5% *w*/*w* mixture of spiked ground chicken in a beaker as prepared as described above was cooked by immersing the beakers (covered with aluminum foil) in boiling water for 15 min. The cooked samples were broken down into finer particles, 20 mL of 10 mM PBS was added, and the mixture was homogenized for 2 min at 11,000 rpm using the ULTRA-TURRAX T25 basic homogenizer (IKA Works Inc., Wilmington, NC, USA). The homogenized samples were then stored, centrifuged, and passed through filter as described above to obtain 5% *v*/*w* spiked cooked chicken meat extracts. Lower levels of spiking (0.01%, 0.03%, 0.05%, 0.1%, 0.3%, 0.5%, 1% and 3% *v*/*v*) were obtained by diluting 5% *w*/*w* spiked samples with the appropriate amount of non-spiked cooked chicken meat (0%) that had been similarly prepared. All Spiked sample extracts were prepared on the same day and tested immediately after preparation. Ground beef and pork spiked with different amounts of RBC-containing product (PHP, APS, APT or APR) were prepared for studies on matrix effect as described above for spiked ground chicken.

### 2.2. Non-Competitive Indirect Enzyme-Linked Immunosorbent Assay (iELISA)

The selectivity of mAb 24C12-E7 was determined using antigen-coated indirect non-competitive iELISA as previously described [1] with modifications as follows. Plates were incubated for 1 h at 37 °C; 0.2% fish gelatin in 10 mM PBS and PBST (0.05% *v/v* Tween-20 in 10 mM PBS) used as blocking and antibody buffer respectively; and absorbance read at 415 nm using the PowerWave XS microplate reader (Bio-Tek Instruments, Winooski, VT, USA). Sample diluent (0.06 carbonate buffer) was run alongside test samples as blanks and the average absorbance subtracted from readings obtained for test samples. 

### 2.3. Sodium Dodecyl Sulfate–Polyacrylamide Gel Electrophoresis (SDS-PAGE) and Western Blot

SDS-PAGE followed by western blot was performed to reveal the presence of the 12 kDa antigenic protein in various samples. Briefly, soluble proteins (loaded at 2 to 20 μg of protein in 10 μL sample buffer per lane depending on the sample) from the samples were loaded onto 5% stacking gels and separated on 15% polyacrylamide separating gels at 200 V with the aid of the Mini-Protein 3 Electrophoresis Cell (Bio-Rad) as per the method of Laemmli [7]. The separated proteins were transferred electrophoretically (1 h at 100 V, using the Mini Trans-Blot Electrophoretic Transfer Cell, Bio-Rad) according to the protocol by Towbin and others [8] and the membrane blocked with 0.2% fish gelatin in tris buffered saline (TBS). The blotted membrane was then incubated with selected mAb 24C12-E7 followed by incubation with secondary antibody (goat anti-mouse IgG (H + L)-AP conjugate) and subsequent color development as previously described [1]. Precision Plus Protein Kaleidoscope standards were used for the molecular-weight estimations on gels and blot.

### 2.4. Two-Dimensional Gel Electrophoresis and Western Blot

Two-dimensional electrophoresis (TDGE) was carried out on purified porcine hemoglobin (25 μg per 125 μL of rehydrating buffer) with isoelectric focusing (using the PROTEAN IEF Cell, Bio-Rad) as the first dimension and SDS-PAGE as the second dimension as previously described [6]. The gel was subsequently subjected to western blot as described above to determine the isoelectric point of the 12 kDa protein that is recognized by mAb 24C12-E7. 

### 2.5. N-Terminal Sequencing 

Purified porcine hemoglobin that has been subjected to TDGE was first transferred unto a Westran S PVDF (polyvinylidene fluoride) (0.2 μm) membrane as described above. Subsequently, the transferred proteins were stained with EZ-Blue and spot on the PVDF membrane that corresponds to spots on the nitrocellulose membrane that reacted with mAb 24C12-E7, was excised and subjected to *N*-terminal amino acid sequence using an ABI 477A sequencer with an online 120A HPLC system (Applied Biosystems Inc., Foster City, CA, USA). The sequence data was collected using the ABI 610 software (Applied Biosystems Inc.) and analyzed with FASTA programming (European Bioinformatics, http://www2.ebi.ac.uk/fasta3/) 

## 3. Results and Discussion

### 3.1. Identity of the 12 kDa Antigenic Protein 

**Figure 1:**
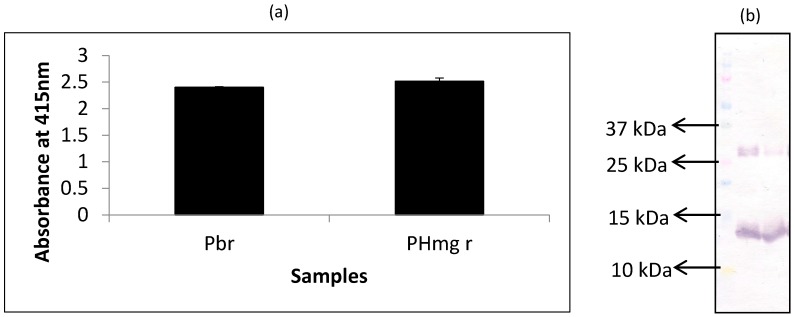
Analysis of raw porcine blood (Pbr) and porcine hemoglobin extracts (PHmg) using (**a**) iELISA and (**b**) western blot using mAb 24C12-E7 as probe. Samples were coated at 0.5 μg per 100 μL of carbonate buffer in iELISA. mAb 24C12-E7 supernatant was diluted 1:50 in 0.2% fish gelatin in PBST and iELISA results are expressed as A_415_ ± SD, *n* = 3 (SD = standard deviation). In the case of western blot samples were loaded at 10 μg per 10 μL of sample buffer alongside the Precision Plus Protein Kaleidoscope pre-stained standards.

In this experiment, extracts of porcine hemoglobin were probed with mAb 24C12-E7 using both iELISA and western blot. Porcine blood was run alongside as the control for comparison. From the results of iELISA (Figure 1a), mAb 24C12-E7 reacted very strongly (OD > 2) with both non-heated porcine blood and porcine hemoglobin extracts. Western blot results (Figure 1b) also corroborates the iELISA results as mAb 24C12-E7 reacted with a 12 kDa in both porcine blood and porcine hemoglobin samples suggesting that this 12 kDa antigenic protein is likely to be a monomer of porcine hemoglobin as anticipated. Both samples also revealed a band at around 26 kDa which could be a dimer of this 12 kDa protein as will be discussed below. 

**Figure 2:**
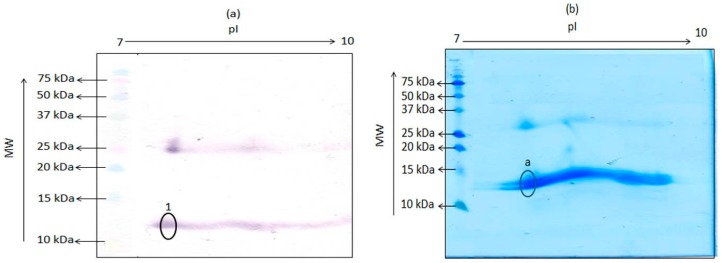
Two-dimensional gel electrophoresis of porcine hemoglobin extract containing 25 μg total protein followed by transfer unto (**a**) nitrocellulose membrane and detection with mAb 24C12-E7 and (**b**) PVDF membrane and subsequent staining with EZBlueTM. Precision Plus Protein Kaleidoscope pre-stained standards were run alongside for estimation of molecular weight of proteins. Circled spot “a” on the PVDF membrane was subjected to *N*-terminal sequencing.

The hemoglobin tetramer is a weak complex of 2 alpha-beta dimers held together by a bond between an alpha and a beta subunit. The beta and alpha subunits were reported to have pIs of 7.1 and 8.76, respectively [9]. Thus, as further proof of the 12 kDa antigenic protein being a monomer of porcine hemoglobin, extract of porcine hemoglobin was subjected to TDGE followed by blotting with mAb 24C12-E7, in order to determine the pI of this porcine 12 kDa protein. As shown in Figure 2a, mAb 24C12-E7 reacted with several poorly separated spots around the molecular weight of 12 kDa and between the pIs of 7.5 and 9.5, instead of two distinct spots with pIs of 7.1 and 8.76. Several such poorly separated spots were also visible at a molecular weight of around 26 kDa. A similar observation was reported previously where TDGE of bovine hemoglobin produced several spots with molecular weight around 12 kDa [6]. Other researchers have also reported the presence of multiple spots upon subjecting hemoglobin from other species to TDGE [10], an observation that has been ascribed to the presence of impurities, dimerization, or variable phosphorylation [11]. Finally, a spot, circled and labeled “a” (Figure 2b) on PVDF, corresponding to the spot circled and labeled “1” (Figure 2a) on the nitrocellulose membrane that reacted with mAb 24C12-E7, was excised and subjected to N-terminal sequencing as a conclusive proof if this 12 kDa antigenic protein is indeed a monomer of the tetrameric hemoglobin molecule. Results of the sequencing revealed that the spot was a mixed sequence of two overlapping spots with the first 12 amino acids of both sequence as shown in Table 1. Sequences 1 and 2 showed 100% homology with the alpha and beta subunit of porcine hemoglobin, respectively, when the sequences obtained was blasted using the NCBI Blast: Protein Sequence software (http://blast.ncbi.nlm.nih.gov/Blast.cgi?PAGE=Proteins). These results confirm this 12 kDa protein in porcine blood to be indeed a monomer of the tetrameric hemoglobin molecule.

### 3.2. Selectivity of mAb 24C12-E7 Based iELISA

Having demonstrated that the 12 kDa antigenic protein recognized by mAb 24C12-E7 is a monomer of the tetrameric hemoglobin molecule, a simple iELISA was optimized and developed to demonstrate the utility of mAb 24C12-E7 for monitoring the presence of porcine blood or porcine RBC-derived proteins in foods based on its reaction with the 12 kDa protein. The first in the series of experiments was to determine the ability of the iELISA to discriminate (1) between porcine blood and blood from other species; (2) porcine RBCs from porcine plasma; and (3) porcine blood from non-blood proteins that are likely to be present in a food matrix. 

**Figure 3:**
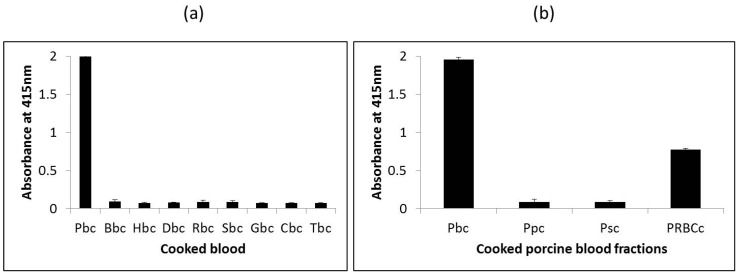
Reactivity of mAb 24C12-E7 with (**a**) cooked blood extracts from nine species and (**b**) cooked porcine blood and blood fractions using iELISA. Soluble proteins extracted from cooked whole blood, plasma, serum or RBCs (red blood cells) were coated at 0.5 μg per 100 μL. mAb 24C12-E7 supernatant was diluted 1:50 in 0.2% fish gelatin in PBST . Results are expressed as A_415_ ± SD, *n* = 3. Pbc: cooked porcine blood; Bbc: cooked bovine blood; Hbc: cooked horse blood; Dbc: cooked donkey blood; Rbc: cooked rabbit blood; Sbc: cooked sheep blood; Gbc: cooked goat blood; Cbc: cooked chicken blood; Tbc: cooked turkey blood; Ppc: cooked porcine plasma; Psc: cooked porcine serum; and PRBCc: cooked porcine RBCs.

The ability of the assay to distinguish porcine blood from other species was determined by testing the assay against porcine blood and blood from other species, including bovine, horse, donkey, goat, sheep, rabbit, chicken and turkey. Since commercially produced blood proteins typically undergo heat-treatment by way of spray-drying, soluble proteins extracted from heated blood from the various species were used as samples for testing. As shown in Figure 3a, the assay reacted strongly with heated porcine blood (A_415_ = 2.0) but did not react with heated blood from the other eight species tested (A_415_ < 0.1) indicating the porcine selectivity of this mAb 24C12-E7 and the heat-stability of its epitope. Subsequently, analysis of extracts of heated porcine blood and porcine blood fractions (porcine serum, porcine plasma and porcine RBCs) revealed the assay to be specific for porcine RBC as the assay reacted with porcine blood (A_415_ = 1.95) and porcine RBCs (A_415_ = 0.78), but not with porcine plasma (A_415_ < 0.1) or porcine serum (A_415_ < 0.1). The much lower reading for porcine RBCs in comparison with porcine blood is attributed to the much lower amounts of the 12 kDa antigenic protein (hemoglobin) present in the former which was purchased as a 10% diluted solution.

The mAb 24C12-E7 based iELISA was further tested against extracts prepared from a number of common food proteins (whey, soy, egg albumin, non-fat dry milk, bovine serum albumin, and porcine gelatin) and the meat of thirteen species (pig, cow, deer, elk, horse, African buffalo, rabbit, lamb, bison, chicken, turkey, goose and duck) for examining the assay’s cross-reactivity. Results clearly showed that the assay did not cross-react (A_415_ < 0.1) with any of these non-blood proteins tested. The results demonstrated the excellent selectivity of this mAb24C12-E7 based assay. 

### 3.3. Reactivity of the iELISA with Commercial Blood Proteins 

**Figure 4:**
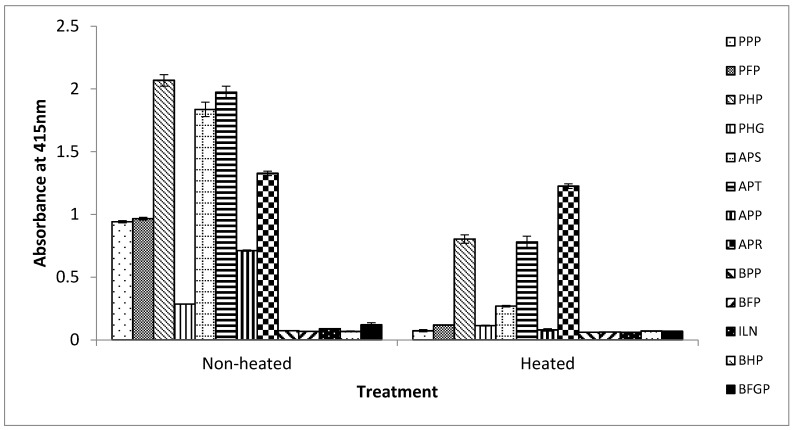
Reactivity of mAb 24C12-E7 with non-heated and heated bovine and porcine blood-derived food ingredients using iELISA. Soluble proteins extracted from these blood-derived proteins were coated at 0.5 μg/100 μL. mAb 24C12-E7 supernatant was diluted 1:50 in 0.2% fish gelatin in PBST. Results are expressed as A_415_ ± SD, *n* = 3. PPP: porcine plasma powder; PFP: porcine Fibrimex^®^ powder; PHP: porcine hemoglobin powder; PHG: porcine hydrolyzed globin; APS: Aprosan; APT: Aprothem; APP: Apropork; APR: Aprored; BPP: bovine plasma powder; BFP: bovine Fibrimex^®^ powder; ILN: Immunolin^®^; BHP: bovine hemoglobin powder; and BFGP: bovine fibrinogen powder.

From the results above, this iELISA based on the detection of the 12 kDa antigenic protein in porcine RBCs has potential for accurately detecting the presence of raw and processed porcine blood and porcine RBC-derived proteins in foods. Therefore, the assay was tested against commercially produced spray-dried porcine and bovine blood proteins that are obtained either from whole blood, or from the plasma or RBC fraction. Non-heated and heated (further heated by immersing in boiling water for 15 min) versions of these spray-dried proteins were analyzed. Heated versions of these proteins were included to examine how the assay will perform in situations where they are used as ingredients in foods that may undergo further heat-treatment in their production. As shown in Figure 4, for the non-heated spray-dried products, the assay did not react with any of the bovine blood proteins (bovine plasma powder (BPP), bovine hemoglobin powder (BHP), bovine Fibrimex^®^ powder (BFP), Immunolin^®^ (ILN), and bovine fibrinogen powder (BFGP)) (A_415_ < 0.1) tested as expected (Figure 4). In the case of the porcine proteins, the assay reacted strongly with the porcine RBC-derived proteins, namely, porcine hemoglobin powder (PHP) (A_415_ = 2.07), Aprothem (APT) (A_415_ = 1.97), and Aprored (APR) (A_415_ = 1.33) but weakly with porcine hydrolyzed globin (PHG) (A_415_ = 0.286). Apparently, the weak reaction with PHG is due to destruction of the epitope by the hydrolysis involved in the production of this product. The assay also exhibited a strong reaction to the whole porcine blood product, Aprosan (APS) (A_415_ = 1.84). However, the assay also unexpectedly reacted moderately with the two porcine plasma-derived proteins, porcine plasma powder (PPP) (A_415_ = 0.94) and porcine Fibrimex^®^ powder (PFP) (A_415_ = 0.97). Based on the excellent porcine hemoglobin selectivity of this assay demonstrated in the above section, this positive reaction obviously can be attributed to the poor quality control practice of the commercial manufacturing by introducing contamination with hemoglobin either from the poor separation of the plasma from the red blood cells, or as a result of cross contamination with porcine RBC-derived proteins that are also produced by the same company. 

For the further heated protein samples, the overall reaction signals were reduced compared to the non-heated spray-dried samples. The assay neither reacted with the bovine products (BPP, BFP, BHP, ILN, and BFGP) (A_415_ < 0.1) nor with any of the porcine plasma-derived proteins (PPP, PFP, and APP) (A_415_ < 0.1) but reacted only with the porcine RBC-containing proteins, namely, PHP (A_415_ = 0.8), APT (A_415_ = 0.78), APR (A_415_ = 1.23), and APS (A_415_ = 0.27), except in the case of the hydrolyzed product, PHG (A_415_ < 0.1) (Figure 4). According to the manufacturers, these spray-dried protein products were already heat-processed to internal temperatures of 70 °C or 80 °C. Thus, in the case of the heated samples, the further heat-treatment may have destroyed and insolubilized significant amount of the antigenic proteins, and hence the much lower reaction signals. For those proteins containing porcine RBCs, the reduction in signals (absorbance) for non-heated versions compared to heated versions were 61%, 60%, 85%, 60%, and 7% for PHP, PHG, APS, APT, and APR, respectively. This may explain the lack of binding with the hemoglobin contaminated porcine plasma-derived proteins in the case of the heated versions, as the further heat-treatment (boiling for 15 min) may have diminished any contaminated antigenic protein present to levels below the detection limit of the assay. 

### 3.4. Effect of Heat-Treatment on the 12 kDa Antigenic Protein 

It is apparent that the epitope of 12 kDa protein recognized by mAb 24C12-E7 may be affected when the protein is subjected to excessive heat processing which consequently may affect its detectability using ELISA. Accordingly, extracts of these porcine blood proteins that are derived from both plasma and RBCs were further analyzed with western blot to observe the changes of this antigenic protein. Porcine plasma-derived proteins were included as negative controls. 

**Figure 5:**
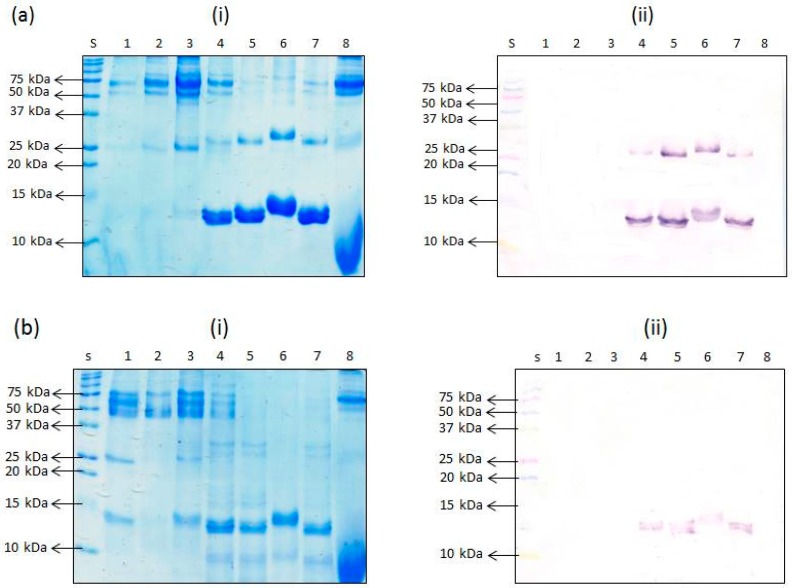
SDS PAGE (**i**) and western blot (**ii**) analysis of non-heated (**a**) and heated (**b**) extracts of porcine blood-derived proteins. Protein extracts were loaded at 10 μg per 10 μL of sample buffer per lane alongside Precision Plus Protein kaleidoscope pre-stained standards. Lane s: Precision Plus Protein Kaleidoscope standards; Lane 1: porcine plasma powder (PPP); Lane 2: porcine Fibrimex^®^ powder (PFP); Lane 3: Apropork (APP); Lane 4: Aprosan (APS); Lane 5: porcine hemoglobin powder (PHP); Lane 6: Aprored (APR); Lane 7: Aprothem (APT); and Lane 8: porcine hydrolyzed globin (PHG).

In the case of the non-heated proteins, SDS-PAGE revealed the presence of the 12 kDa proteins in the porcine RBC-derived proteins, PHP (lane 5) and APT (lane 7), and the whole porcine blood product, APS (lane 4). In the case of the other RBC-derived product, APR (lane 6), a slightly higher band at around 13 kDa was revealed (Figure 5a(i)). APR is a branded product with scarce information about its nature except for the disclosed fact that it is a pigment obtained from porcine RBCs. Perhaps modifications to the hemoglobin by the processing involved in its production may have caused it to move at a slightly slower rate and hence the 13 kDa band observed instead of the 12 kDa band. The 12 kDa band, however, was absent in the hydrolyzed product, PHG (lane 8). It appears that the hydrolysis action breaks down the protein into lower molecular weight peptides which can be seen as a smear below 10 kDa. A band at around 25 kDa was also present in the samples, PHP, APT, and APS, and also present faintly in PHG (Figure 5a(i)). A slightly higher band at around 27 kDa was present in APR. Other protein bands (>50 kDa) were present in all of these samples (PHP, APT, APS, APR, and PHG) (Figure 5a(i)). When these proteins were blotted and probed with mAb 24C12-E7, the antibody reacted with the 12 kDa protein and the 25 kDa protein bands in PHP, APT, and APS, and the corresponding slightly higher bands at 13 kDa and 27 kDa, in the case of APR (Figure 5a(ii)). It is reasonable to consider that the 25 or 27 kDa antigenic proteins are a dimer of the 12 and 13 kDa proteins, respectively. Lack of binding with the higher molecular weight bands (>50 kDa) revealed on SDS-PAGE may be because these proteins are non-specific contaminants. The control samples, porcine plasma-derived proteins PPP (lane 1), PFP (lane 2) and APP (lane 3) also revealed the 12 kDa band in increasing order of intensity, although much fainter in comparison with RBC-containing products (PHP, APS, APT and APR). This corroborates our earlier assertion that the observed reaction with non-heated porcine plasma proteins (PPP, PFP and APP) using iELISA was due to contamination with porcine hemoglobin. Interestingly, unlike in the case of iELISA, mAb 24C12-E7 did not react with the 12 kDa protein in these plasma-derived samples. Studies have shown that the mean total quantity of all proteins transferred onto the western blot membrane over a 2 h period using conventional tank transfer (which was employed in this study) is 57% [11]. Thus, the little amount of contaminant protein residues, judging from the faint to almost indiscernible bands, could have easily been lost during the transfer or diminished to levels below the Western blot detection limit (Figure 5a(ii)).

From the SDS-PAGE results for the heat-treated counterparts, a similar observation as the iELISA was evident showing overall weaker intensity of the 12 kDa band in the RBC-containing products, namely, APS (lane 4), PHP (lane 5), and APT (lane 7) (Figure 5b(i)). Likewise, the slightly heavier band (13 kDa) in the product APR was also weaker in intensity compared to its non-heated counterparts. The band intensity for the dimers (25 or 27 kDa proteins) were even weaker or absent in the product APR (lane 6). Upon subsequent transfer and blotting with mAb 24C12-E7, the 12 kDa protein also appeared in the heated products, APS, PHP and APT, and the 13 kDa protein in APR, but with much weaker band intensities compared to the non-heated versions. The protein bands revealed on SDS-PAGE for the porcine plasma-derived protein samples were also not revealed by Western blot analysis (Figure 5b(ii)) due to overall heat-induced (from spray-drying, cooking for 15 min and sample heat-treatment for 5 min prior to SDS-PAGE) reduction in binding between mAb 24C12-E7 and its 12 kDa antigen. 

In summary, from the submissions made above, it is apparent that excessive heat treatment as in spray-drying followed by subsequent heating for 15 min, affects the integrity of the antigenic protein in a manner that diminishes its detectability.

### 3.5. Detection Limit of mAb 24C12-E7 Based iELISA 

**Figure 6:**
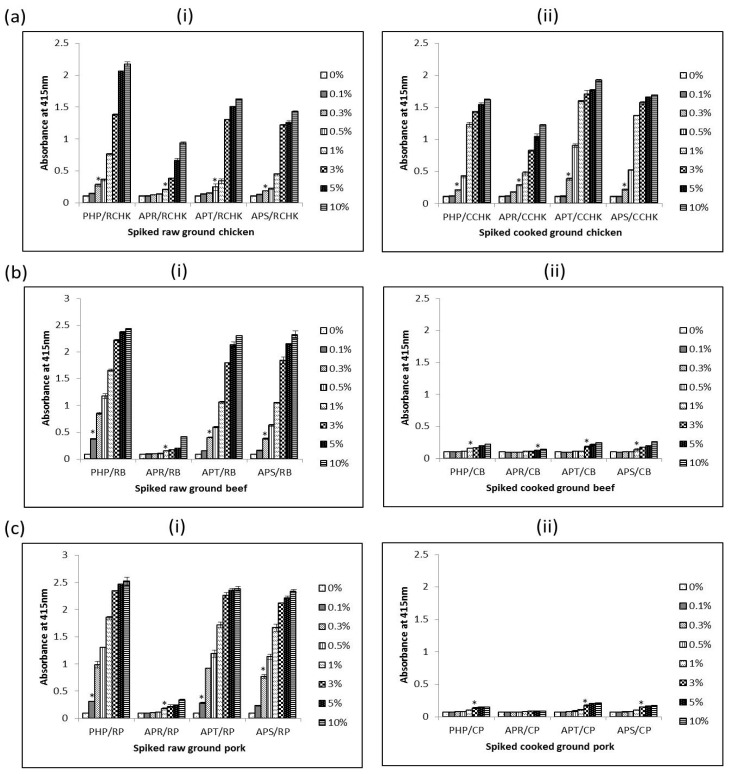
Detection limit of PHP, APR, APT, and APS in raw (**i**) and cooked (**ii**) ground chicken (**a**), ground beef (**b**) and ground pork (**c**) using 24C12-E7 based iELISA. Results are expressed as A_415_ ± SD, *n* = 3. An asterisk (*) indicates a significant difference (*p* < 0.05) from background (0%). RCHK: raw ground chicken; CCHK: cooked ground chicken; RB: raw beef; CB: cooked beef; RP: raw pork; and CP: cooked pork.

The interference of using PRBCIs in various food matrices was investigated by testing both raw and cooked meat products spiked with different amounts of each PRBCI (PHP, APT, and APR) and porcine whole blood protein (APS). The detection limits of porcine materials in different food matrices were determined using the iELISA. The detection limit of the assay was defined as the minimum amount of spiked sample that was statistically different from un-spiked meat (0%) samples. As summarized in Table 2, the assay could sensitively detect 0.3%, 1%, 0.5% and 0.3% (*v*/*v*) of porcine hemoglobin powder (PHP), Aprored (APR), Aprothem (APT), and Aprosan (APS) in raw ground chicken, respectively (Figure 6a(i))). Surprisingly, for spiked cooked ground chicken samples, the detection limits were even lower in some cases as in 0.3% (*v*/*v*), 0.5% (*v*/*v*), 0.3% (*v*/*v*) and 0.3% (*v*/*v*) PHP, APR, APT, and APS in cooked ground chicken, respectively (Figure 6a(ii)). In the case of spiked ground beef, the detection limit for the assay was 0.1% (*v*/*v*), 1% (*v*/*v*), 0.3% (*v*/*v*) and 0.3% (*v*/*v*) PHP, APR, APT, and APS in raw spiked ground, respectively (Figure 6b(i)); and 1.0% (*v*/*v*), 5.0% (*v*/*v*), 3.0% (*v*/*v*), and 1.0% (*v*/*v*) PHP, APR, APT, and APS in cooked spiked counterparts, respectively (Figure 6b(ii)). The assay could also detect 0.1% (*v*/*v*), 1.0% (*v*/*v*), 0.1% (*v*/*v*), and 0.3% (*v*/*v*) of PHP, APR, APT, and APS in raw ground pork respectively (Figure 6c(i)). For spiked cooked ground pork, 3.0% (*v*/*v*) of PHP, APT, and APS could be detected (Figure 6b(ii)). However, in the case of cooked pork spiked with APR, the assay could not detect APR up to inclusion levels of 10% (*w*/*w*) (Figure 6b(ii)).

From the results, it is obvious that different matrices have different effects on the measurability of the 12 kDa antigen. Despite the differences, for raw samples the assay could sensitively detect 1.0% (*v*/*v*) or less of these porcine RBC-derived or porcine whole blood proteins in all spiked meat samples. In the case of cooked spiked samples, heating affects the antigen in a manner that reduces its immunoreactivity as seen with higher readings of non-heated proteins in comparison with heated versions. The detection limits were several times higher than those of their raw spiked sample counterparts. This observation, however, seems to apply to red meats (beef and pork) only, as the detection limits for cooked spiked samples in chicken meat were comparable to those of the raw samples. The higher detection limit for spiked cooked sample is understandable as these proteins had already undergone heat-treatment by way of spray-drying and the extra heat treatment (boiling in water for 15 min) can further destroy the epitope leading to a lower immunoreactivity (and hence higher detection limits) for cooked samples compared to spiked raw meat samples. It is unclear why cooking of spiked ground chicken did not affect detectability of the antigenic protein as was the case with cooked spiked ground beef and pork. Perhaps some components in the ground chicken sample may have protected the 12 kDa protein from the effects of heat. 

## 4. Conclusions

In conclusion, the 12 kDa antigenic protein recognized by mAb 24C12-E7 has been identified as a monomer of the tetrameric hemoglobin molecule based on the immunoreactivity, molecular size, relative heat-stability and amino acid analysis. Further studies have also shown that in situations where this antigenic protein has previously been subjected to severe processing as in spray-drying at internal temperatures of 70 °C or 80 °C, followed by cooking in boiling water for 15 min, its integrity is affected in a manner that makes it less detectable. Consequently, the developed iELISA using mAb 24C12-E7 could detect 1% (*v*/*v*) or less of diversely processed porcine hemoglobin-containing food ingredients in raw ground meats. However, for spiked cooked samples, except for cooked spiked chicken where the assay could detect 0.5% (*v*/*v*) or less of these porcine blood proteins, the detection limits were 3 to 10 times higher for cooked spiked beef, and even higher (≥30 times) for cooked spiked pork. These results demonstrate that the mAb 24C12-E7 based iELISA has the potential for use as an analytical tool for monitoring the presence of spray-dried porcine hemoglobin-containing blood protein ingredients in foods for the protection of those that avoid consuming blood for various reasons. However, in situations where these spray-dried proteins have been used in formulations that have undergone further heat-processing, the assay may not be suitable when these proteins have been included at levels below 3% or even 10% for some of these proteins.

## Figures and Tables

**Table 1:** *N*-terminal sequence data for the excised spot labeled “a” on PVDF (polyvinylidene fluoride).

Sequence	First 12 Amino Acids
Sequence 1	V L S A A D K A N V K A
Sequence 2	V H L S A E E K E A V L

**Table 2:** Summary of detection limit of spiked meat samples using 24C12-E7 based iELISA.

Spiked Samples	Detection Limit	
	Raw	Cooked
1. Porcine hemoglobin powder in ground chicken	0.3% (*v*/*v*)	0.3%
2. Aprored in ground chicken	1.0% (*v*/*v*)	0.5%
3. Aprothem in ground chicken	0.5% (*v*/*v*)	0.3%
4. Aprosan in ground chicken	0.3% (*v*/*v*)	0.3%
1. Porcine hemoglobin powder in ground beef	0.1% (*v*/*v*)	1% (*v*/*v*)
2. Aprored in ground beef	1.0% (*v*/*v*)	5.0% (*v*/*v*)
3. Aprothem in ground beef	0.3% (*v*/*v*)	3.0% (*v*/*v*)
4. Aprosan in ground beef	0.3% (*v*/*v*)	1.0% (*v*/*v*)
1. Porcine hemoglobin powder in ground pork	0.1% (*v*/*v*)	3.0% (*v*/*v*)
2. Aprored in ground pork	1.0% (*v*/*v*)	Undetectable up to 10.0% (*w*/*w*)
3. Aprothem in ground pork	0.1% (*v*/*v*)	3.0% (*v*/*v*)
4. Aprosan in ground pork	0.3% (*v*/*v*)	3.0% (*v*/*v*)

## References

[1] Hsieh Y.H., Ofori J.A., Rao Q., Bridgeman C.R. (2007). Monoclonal antibodies specific to thermostable proteins in animal blood. J. Agric. Food Chem..

[2] Ofori J.A., Hsieh Y.H. (2015). Characterization of a 60-kDa Thermally Stable Antigenic Protein as a Marker for the Immunodetection of Bovine Plasma-Derived Food Ingredients. J. Food Sci..

[3] Ofori J.A., Hsieh Y.H. Immunodetection of bovine hemoglobin-containing food ingredients using monoclonal antibody Bb 1H9. Food Control.

[4] Raja Nhari R.M., Hamid M., Rasli N.M., Omar A.R., El Sheikha A.F., Mustafa S. (2016). Monoclonal antibodies specific to heat-treated porcine blood. J. Sci. Food Agric..

[5] Ofori J.A., Hsieh Y.H. (2016). Monoclonal Antibodies as Probes for the Detection of Porcine Blood-Derived Food Ingredients. J. Agric. Food Chem..

[6] Ofori J.A., Hsieh Y.H. (2011). Characterization of a 12 kDa thermal-stable antigenic protein in bovine blood. J. Food Sci..

[7] Laemmli U.K. (1970). Cleavage of structural proteins during the assembly of the head of bacteriophage T4. Nature.

[8] Towbin H., Staehelin T., Gordon J. (1979). Electrophoretic transfer of proteins from polyacrylamide gels to nitrocellulose sheets: Procedure and some applications. Proc. Natl. Acad. Sci. USA.

[9] Zhang K., Liu Y., Shang Y., Zheng H., Guo J., Tian H., Jin Y., He J., Liu X. (2012). Analysis of pig serum proteins based on shotgun liquid chromatography-tandem mass spectrometry. Afr. J. Biotechnol..

[10] Xu L., Glatz C.E. (2009). Predicting protein retention time in ion-exchange chromatography based on three-dimensional protein characterization. J. Chromatogr. A.

[11] Tovey E.R., Baldo B.A. (1987). Comparison of semi-dry and conventional tank-buffer electrophoresis of proteins from polyacrylamide gels to nitrocellulose membranes. Electrophoresis.

